# Sweet Malaria

**Published:** 1886-08

**Authors:** 


### CULLINGS FROM CONTEMPORARIES

### SWEET MALARIA.

Malaria, Malaria,
Sweet, loving, true, Malaria,
Scott Way and naughty R. K.
Have slandered thee, Malaria !
But we are friends—I love thee well.
What tongue or lips can ever tell
The fun we've found in swamp and dell
'Mid Jersey's watery area?
Gentle archer—merry shaker—
Patron of the undertaker—
I sing thy praise, Malaria !

Ah! faithful friend, Malaria,
'Twas not in far Bavaria,
Nor Hunter's Point nor Harlem Flats,
Nor Jersey's vast aquaria,
That first I shook in terror wild
Till buildings seven stories piled,
Tottered and trembled like a child,
Half frantic with hysteria!
Nay - I solicit for my deficit
Pardon that I have chanced to miss it—
I've never had malaria!

Then why, O sweet Malaria,
Sing I thy praise, if nary a
Shake I've had in fields of rye
And fragrant Antennaria?
Ah! "thereby hangs a tale." You see,
When first you stood revealed to me,
Your victim paid his doctor's fee
To me, my dear Malaria;
For my position is physician
To those who have malaria;
To peddle quinine is my mission;
And, since thou betterest my condition,
I love thee, sweet Malaria,
Sweet, loving, true Malaria!

—*Ala. Med. Journal.*

